# Plasma Treating Mixed Metal Oxides to Improve Oxidative Performance via Defect Generation

**DOI:** 10.3390/ma12172756

**Published:** 2019-08-27

**Authors:** Jonathan Horlyck, Alimatun Nashira, Emma Lovell, Rahman Daiyan, Nicholas Bedford, Yuexing Wei, Rose Amal, Jason Scott

**Affiliations:** 1 School of Chemical Engineering, The University of New South Wales, Sydney, NSW 2052, Australia; 2 School of Energy and Environment, Southeast University, Nanjing 210096, China

**Keywords:** silica-titania photocatalysts, defect generation, plasma pre-treatment, oxygen activation

## Abstract

The generation of structural defects in metal oxide catalysts offers a potential pathway to improve performance. Herein, we investigated the effect of thermal hydrogenation and low-temperature plasma treatments on mixed SiO_2_/TiO_2_ materials. Hydrogenation at 500 °C resulted in the reduction of the material to produce Ti^3+^ in the bulk TiO_2_. In contrast, low temperature plasma treatment for 10 or 20 min generated surface Ti^3+^ species via the removal of oxygen on both the neat and hydrogenated material. Assessing the photocatalytic activity of the materials demonstrated a 40–130% increase in the rate of formic acid oxidation after plasma treatment. A strong relationship between the Ti^3+^ content and catalyst activity was established, although a change in the Si–Ti interaction after plasma treating of the neat SiO_2_/TiO_2_ material was found to limit performance, and suggests that performance is not determined solely by the presence of Ti^3+^.

## 1. Introduction

Noble metal-free catalysts have gained increasing attention as alternatives for a range of oxidation processes, such as the oxidation of carbon monoxide [1,2], methanol [3,4], toluene [5,6], and formaldehyde [7,8]. While metal oxide catalysts, such as TiO_2_ and SiO_2_, have the key advantage of being cheap and abundant, their performance towards catalytic oxidation remains lower than materials containing noble metals, such as Pt. Thus, improving the catalytic activity of metal oxides via tailored synthesis and pre-treatment methods is of significant interest.

The generation of defects in metal oxides has been shown to enhance numerous catalytic processes, for instance, in water splitting and charge storage [9], the oxygen reduction reaction [10], and carbon dioxide reduction [11]. While defects such as oxygen vacancies are induced naturally during the synthesis of many metal oxides, they can also be generated and altered via treatments such as high-temperature reduction [12,13], doping [14], and illumination [15]. From a photocatalysis perspective, the generation of surface defects in metal oxides acts to provide active sites for reactions, through the ability to trap holes or electrons, resulting in charge separation and facilitating oxygen activation [16,17].

TiO_2_ is a reducible metal oxide which has been shown to readily form surface defects, such as Ti^3+^, via various pre-treatment methods [12,18]. In a previous study, defects in TiO_2_ prepared via flame spray pyrolysis (FSP) were induced by doping with SiO_2_, treating with H_2_ at 500 °C, followed by UV-light pre-illumination [19]. The various pre-treatment methods led to the creation of Ti^3+^ in TiO_2_ and non-bridging oxygen hole centre (NBOHC) defects in SiO_2_. Ultimately, a synergism between these two defects, which was found to be dependent on the Si/Ti ratio, governed catalytic performance towards the oxidation of formic acid. Similar to high temperature hydrogenation, plasma treatment can be used to modify a metal oxide, however, the generation of defects via plasma treatment occurs primarily at the material surface, rather than in the bulk [20]. The highly energetic discharge particles can modify the surface in a short time, especially compared to time-consuming thermal treatments, via charge transfer, sputtering, or deposition [21]. Reported research concerning the plasma treatment of TiO_2_-based materials primarily utilises high temperature or high power plasma [13,22,23,24]. In comparison, there is a distinct lack of knowledge regarding the effect of low-temperature plasma treatments on defect generation and the ensuing influence this has on the catalytic performance of mixed metal oxide systems, such as SiO_2_/TiO_2_. Furthermore, there is new knowledge to be gained by studying what effect using multiple defect-generating treatment methods in combination has on the properties of the mixed metal oxides.

In this work, we studied the generation of defects by the hydrogenation at 500 °C and/or low temperature plasma treatment of a mixed SiO_2_/TiO_2_ composite that was synthesized via FSP. The formation of Ti^3+^ defects during the hydrogenation and plasma treatments was systematically investigated with X-ray photoelectron spectroscopy (XPS), electron paramagnetic resonance (EPR), X-ray absorption spectroscopy (XANES, and EXAFS) analyses, with a link between material pre-treatment and the photocatalytic oxidation of formic acid established.

## 2. Experimental

### 2.1. Mixed Metal Oxide Preparation

Titanium isopropoxide (TTIP, Sigma Aldrich, St. Louis, MO, USA) and hexamethyldisiloxane (HMDSO, Sigma Aldrich) in absolute ethanol were mixed with molar concentration of 1.194 M and 0.066 M, respectively, to give a Si:Ti molar ratio of 1:9. The precursor mixture was then used to prepare a mixed SiO_2_/TiO_2_ material (denoted as SiTi) with an FSP setup, which has been described previously [25]. After synthesis, the SiTi was hydrogenated in a tube furnace (Carbolite, HST 12/400, 2000 W, Hope Valley, UK) by heating to 500 °C at a rate of 5 °C/min under a 50 mL/min flow of 10% H_2_ (N_2_ balance). These conditions were maintained for 3 h before being cooled to ambient temperature. The hydrogenated samples are denoted as SiTi(H).

The plasma treatment was performed at ambient temperature in a dielectric barrier discharge (DBD) plasma reactor (23 W), consisting of a reaction kettle (8 mm height, DBD-100, Corona Lab, Nanjing, China), discharge equipment (DBD-50), and a CPT-2000K plasma generator with a frequency range of 5–20 kHz. For each treatment, a 120–150 mg sample was spread over the middle of the reactor kettle. He (Coregas, >99.999%) with a flowrate of 30 mL/min was introduced into the reactor through insulated metal tubing and converted into a stable plasma upon entering the discharge area (50 mm diameter). The net power used to generate the plasma was 15.0 + 0.1 W. Plasma treatments of 5, 10, 20, and 30 min were used on the neat and hydrogenated SiTi samples, which are denoted as SiTi(*x*) or SiTi(H*x*), respectively, where *x* represents the plasma treatment time.

### 2.2. Catalyst Characterization

The catalyst specific surface area (SSA) was determined using a Micrometritics TriStar 3030 analyzer (Norcross, GA, USA) and calculated by the Brunauer-Emmett-Teller (BET) method. Samples were degassed at 150 °C for 3 h under vacuum prior to analysis. Sample crystallinity was determined by XRD analysis using a PANalytical Xpert multipurpose X-ray diffraction (MPD) instrument (Malvern, UK). The data was collected over an incident beam (Cu K_α1_) angle of 2θ from 10° and 90°, with a step size of 0.026°.

UV-visible spectra were measured using a Shimadzu UV-3600 UV-vis NIR spectrophotometer (Columbia, MD, USA) with BaSO_4_ (Sigma-Aldrich, 99%) as the reference. A light beam with a wavelength range of 200–800 nm was passed over the solid sample at a scan speed of 200 nm min^−1^. FTIR spectra were obtained using a PerkinElmer FTIR Spectrometer/Microscope (Waltham, MA, USA). The sample was exposed to infrared radiation with a wavenumber of 650 to 4000 cm^−1^ and the transmittance was recorded by PerkinElmer Spotlight 400 imaging system (Waltham, MA, USA), with a resolution of 4 cm^−1^.

Raman spectroscopy was carried out using a Renishaw inVia Raman microscope (Wotton-under-Edge, UK) in backscattering configuration, using an Ar laser to yield an excitation line of 514.5 nm. Electron paramagnetic resonance (EPR) spectra of the samples were measured at the microwave frequency of 9.42 GHz (X-band) and power of 2 mW in a Bruker EMX-plus X-band EPR spectrometer. The amplitude was modulated at 5 G and the temperature set to 120 K.

X-ray photoelectron spectroscopy (XPS) was conducted using a Thermo Scientific ESCALAB250Xi (Waltham, MA, USA). Monochromatic Al Kα (1486.68 eV) radiation, with an emission power of 164 W, was used as the source. The Ti2p, Si2p, and O1s core level spectra were recorded and referenced to the surface carbon C1s peak at 285 eV.

X-ray absorption spectroscopy (XAS) measurements were performed at the 10-BM beamline of the advanced photon source, Argonne national laboratory (Lemont, IL, USA). Prepared powders were loaded into 1 mm OD Kapton capillaries and measured in a transmission geometry. Anatase and rutile spectra were recorded using pure Sigma Aldrich and PC-50 (Millennium) nanoparticle samples, respectively. All data processing and analysis was performed using the Demeter XAS software package [26].

### 2.3. Catalyst Activity Testing

Photocatalytic formic acid oxidation was carried out in a reactor setup, as described previously [27], and comprised a glass spiral reactor that encircled a UV lamp (NEC, black light, 365 nm max emission, Minato, Japan). The catalyst mixture was prepared by adding 50 mg of SiTi catalyst to 50 mL of Milli-Q water and sonicating for 15 min to ensure uniform dispersion within the suspension. Perchloric acid (0.5 M) was added dropwise to the solution until a pH of 3.0 + 0.1 was reached. The catalyst suspension was then added to the spiral reactor, where it was circulated using a peristaltic pump. The UV lamp was switched on for 30 min to eliminate organic matter in the mixture and ensure that the lamp intensity was constant. After switching the lamp off, the injection port was opened for 10 min to allow for equilibration with air, before closing the port once more for a further 10 min. The formic acid oxidation reaction was commenced by switching on the lamp and adding 100 μmol of formic acid. Mineralization of the acid was determined via a change in conductivity (as measured by a Jenway 3540 probe) as a result of carbon dioxide generation. Catalyst activity was compared according to the rate at which the catalyst achieved 50% conversion of the injected formic acid (R_50_).

## 3. Results and Discussion

### 3.1. Effect of Hydrogenation

The N_2_ adsorption–desorption analyses of the neat and hydrogenated SiTi material reveals similar properties. Both samples display a similar type II isotherm and H3 hysteresis loop (Figure S1, Supporting Information). While this is often associated with macroporous materials, it has also been reported to result from mesopores forming between particle aggregates [28]. In the case of FSP-synthesized metal oxides, the formation of sinter bridges in the particle agglomerates affords this mesoporosity [29,30]. This is confirmed by the broad pore size distributions, displayed in Figure S2. SiTi and SiTi(H) display similar BET surface areas (98.7 and 96.4 m^2^/g, respectively), suggesting that the hydrogenation treatment does not impact morphology or particle size.

A colour change from white to a pale blue was observed in the SiTi(H) sample after hydrogenation. This is attributed to the reduction of Ti^4+^ to Ti^3+^ [12,31], generating oxygen vacancies. The blue colour of SiTi(H) remained constant after hydrogenation, suggesting that the addition of SiO_2_ is able to effectively stabilise the Ti^3+^ site. The colour change in the material was further investigated with UV-vis analysis (Figure S3), where the emergence of a broad absorbance band was observed above 400 nm in SiTi(H). The band gap potential remained unchanged (approx. 3.40 eV) after hydrogenation, indicating that the presence of SiO_2_ enlarges the bang gap potential above pure anatase (3.20 eV) and rutile (3.02 eV) TiO_2_ [32], but is not affected by the hydrogenation process.

**Figure 1:**
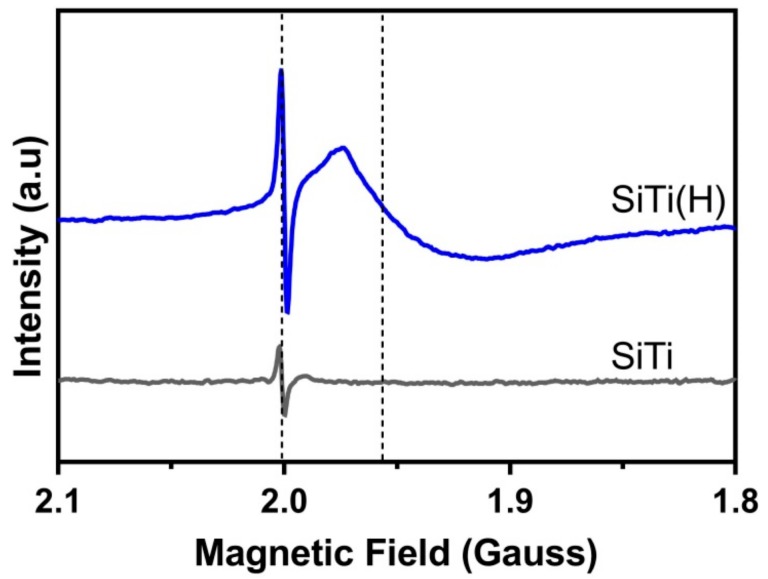
EPR spectra of the FSP-prepared SiTi before and after hydrogenation at 500 °C. Spectra were acquired at 120 K. Dotted lines indicate the presence of defects within the material.

EPR was used to probe the Si/Ti defects generated from hydrogenation, with the results shown in Figure 1. A sharp peak was observed at a g-value of 2.002 in the neat SiTi sample, which indicates that intrinsic E’ centre defects [33] are present in the sample. These are suggested to form as a result of the flame spray pyrolysis synthesis [34]. As flame spray pyrolysis is a rapid process with a very high temperature gradient of cooling, perfect particle rearrangement is not achieved, and E’ centres can be formed. Hydrogenation of the SiTi material did not affect the position of the E’ centre peak, while an increase in peak amplitude for the SiTi(H) suggests that the process may lead to the formation of a greater number of E’ centre defects. A broad peak at a g-value of 1.951 emerges in SiTi(H), with this peak position consistent with the formation of Ti^3+^ defects [19]. This is confirmation that the hydrogenation process is effective in partially reducing Ti^4+^ to Ti^3+^.

**Figure 2:**
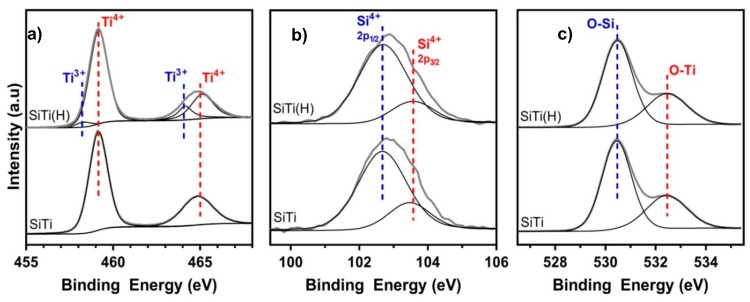
(**a**) Ti2p, (**b**) Si2p, and (**c**) O1s XPS spectra of the FSP-prepared SiTi materials before and after hydrogenation at 500 °C.

The presence of Ti^3+^ defects after hydrogenation was confirmed in the Ti2p XPS spectra (Figure 2a), where the binding energy of the Ti2p_3/2_ and Ti2p_1/2_ peaks (observed at 459.2 eV and 464.9 eV, respectively) remained constant, but two additional peaks at 458.3 eV and 464.2 eV are observed in SiTi(H). Analysis of the separate peaks in the Ti2p XPS spectra allowed for the quantity of Ti^3+^ in the SiTi material to be determined. While neat SiTi shows no sign of Ti^3+^ content being present, as confirmed by EPR and XPS analyses, hydrogenation resulted in a 3.9% content of Ti^3+^ in SiTi(H).

The Si2p spectra contains peaks at 103.5 eV and 102.7 eV, which are attributed to Si^4+^2p_1/2_ and Si^4+^2p_3/2_, respectively. These peak positions are situated at a lower binding energy than what has been reported for SiO_2_ prepared via flame spray pyrolysis [35]. In this case, the shift of Si2p peak binding energy can be attributed to strong interactions between Si and Ti which are formed during synthesis. Hydrogenation of the SiTi material does not appear to affect this interaction, as the peak positions of SiTi(H) and SiTi are identical. This is confirmed in the O1s spectra, where the peak position of the O-Si (530.5 eV) and O-Ti (532.4 eV) are unchanged after hydrogenation.

Hydrogenation of the mixed SiTi material at 500 °C was an effective method of reducing TiO_2_ or Ti^4+^, as evidenced by the change in colour of SiTi(H) to blue and the accompanying broad absorbance above 400 nm in the UV-vis spectra. Furthermore, the generation of Ti^3+^ and accompanying oxygen vacancies is confirmed by the EPR and XPS analyses. Despite the change in Ti properties after hydrogenation, the crystallinity of the bulk material remains unchanged, as shown in Figure S4 (Supporting Information). This, combined with the unchanged relative surface area, shows that hydrogenation is an effective method of targeting changes in the TiO_2_ structure of the SiTi material.

### 3.2. Effect of Plasma Treatment

Plasma treatment offers a method of defect generation that is separate to hydrogenation. It is a low energy method, requiring only 27.6 kJ of energy over the course of the 20 min treatment. Thus, the impact of plasma treatment on the structure and performance of mixed SiO_2_/TiO_2_ materials before and after hydrogenation is assessed.

**Figure 3:**
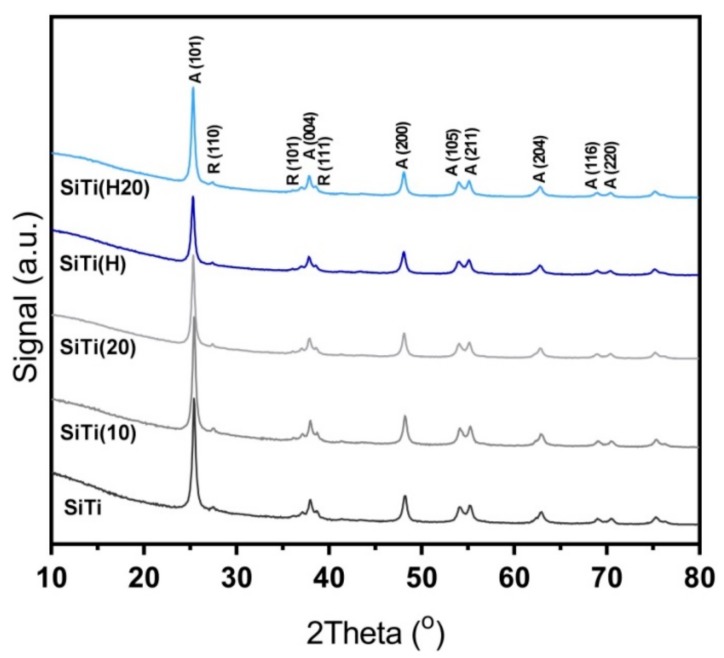
XRD reflections for FSP-synthesized SiTi after hydrogenation and/or plasma treatment. Plasma treatment time denoted in brackets. Anatase (A) and Rutile (R) phases marked.

The X-Ray diffraction pattern (Figure S3) of both the neat SiTi and SiTi(H) sample displays a strong anatase crystal phase. A significantly weaker reflection for the rutile phase was also identified from the small peaks at 27.5°, 36.1°, and 41.4°, which are characteristic of the (110), (101), and (111) planes. The anatase/rutile mixture is consistent with TiO_2_ synthesized via FSP [36]. After plasma treatment for 20 min, no discernible change in peak position or amplitude was observed (as shown in Figure 3), regardless of whether the sample had previously been hydrogenated. This indicates that the anatase/rutile composition and crystallite size of the SiTi is left unchanged in the bulk sample. This does not rule out changes to the surface of the SiTi materials, as XRD does not have sufficient sensitivity to detect such changes, as shown by both Zhuang et al. [37] (for a He plasma system) and Kong et al. [20] (Ar plasma).

**Figure 4:**
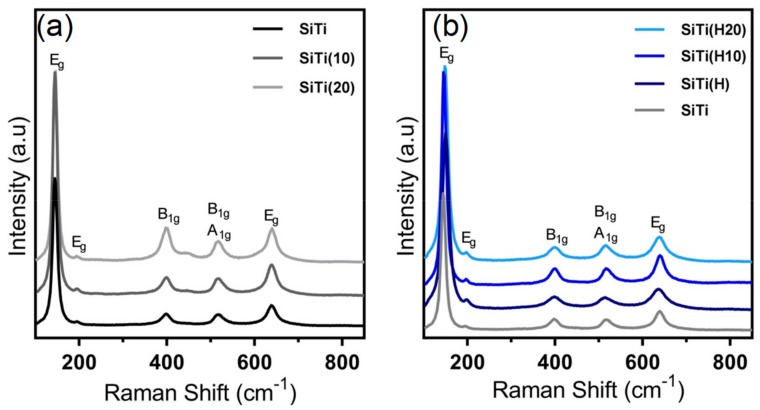
Raman spectra for FSP-synthesized SiTi, after the plasma treatment (with treatment time, in mins, in brackets) of (**a**) neat SiTi and (**b**) hydrogenated SiTi.

To further probe the impact of plasma treatment on the crystal microstructure of SiTi, Raman spectroscopy was carried out on SiTi and SiTi(H) after 0–20 min of plasma treatment (Figure 4). The peak locations in the Raman spectra are in agreement with anatase TiO_2_ [38,39]. The sharp peak at a wavenumber of 144 cm^−1^, shoulder at 195 cm^−1^, and smaller peak at 638 cm^−1^ are attributed to E_g_ symmetry. The peak at 397 cm^−1^ corresponds to the B_1g_ symmetry of anatase, while the peak at 515 cm^−1^ could represent the superposition of both the A_1g_ and B_1g_ symmetry. Plasma treating the neat SiTi sample results in a slight broadening of the peaks and an increase in the ratio of the B_1g_ signal at 397 cm^−1^ to E_g_ signal at 630 cm^−1^. This represents a decrease in local crystallinity as a result of prolonged plasma treatment. An additional peak emerges after 20 min of plasma treatment as a weak shoulder at approximately 440 cm^−1^. This peak position is similar to that reported for the E_g_ phase of rutile [40], but the lack of a corresponding A_1g_ peak at approximately 610 cm^−1^ suggests that there may be another explanation. For example, peak shift and broadening has also been observed for TiO_2_ materials when a surface oxygen deficiency is induced via reduction, where oxygen migrates from surface/subsurface of the TiO_2_ into the bulk [39]. Additionally, a shift or broadening of the TiO_2_ Raman spectra has also been reported when significant internal stress occurs [41]. This will be further explored with XPS analyses of the SiTi materials.

The effect of plasma treatment on SiTi(H) is less pronounced, as shown in Figure 4b. Hydrogenation results in slight peak broadening, potentially representing a decrease in crystallinity and structural symmetry as a result of the formation of Ti^3+^. A similar observation was made for SiTi(H20), but this trend was partially reversed for SiTi(H10), which was plasma treated for 10 min. Unlike the neat SiTi, prolonged plasma treatment of the hydrogenated samples did not result in the emergence of a new peak. This suggests that the reduction of Ti^4+^ to Ti^3+^ via hydrogenation stabilises the TiO_2_ structure in SiTi(H).

**Figure 5:**
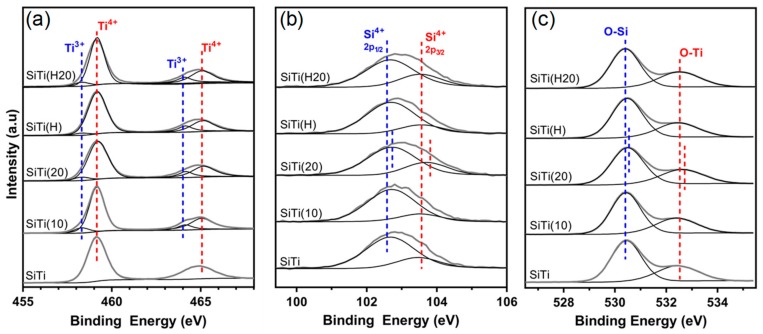
(**a**) Ti2p, (**b**) Si2p, and (**c**) O1s XPS spectra of the neat SiTi and hydrogenated SiTi(H) after plasma treatment for 10 and 20 min.

Analysis of the surface of SiTi and SiTi(H) with XPS before and after plasma treatment revealed that even 10 min of plasma treatment is effective in generating Ti^3+^ defects, as shown in Figure 5a. While the neat SiTi material contained only the Ti^4+^ species, SiTi(10) and SiTi(20) have a Ti^3+^ content of 5.5% and 6.5%, respectively. Similarly, the plasma treatment increased the Ti^3+^ content of the hydrogenated SiTi(H) from 3.9% to 6.8% after a 20 min plasma treatment. Despite SiTi(10) and SiTi(20) being shown via XPS to contain significant Ti^3+^, no colour change to blue was observed. This is explained by the surface nature of the plasma treatment process and low penetration depth of XPS analyses [42]. Low temperature DBD plasma treatments have been shown to be effective in modifying the surface of materials [22,43], rather than the bulk, indicating that the Ti^3+^ content after plasma treatment is likely much higher on the surface of the SiTi materials than in the bulk. As a result, SiTi(10) and SiTi(20) remain white in colour.

The Si2p and O1s XPS spectra (Figure 5b,c, respectively) give an insight into the impact of the plasma treatment on the O and Si species in the SiTi materials. As already discussed, hydrogenation did not result in a shift in the binding energy of the peaks within the Ti, Si, or O spectra. Likewise, plasma treatment of SiTi for 10 min showed no significant peak shifts, however, increasing the treatment time to 20 min resulted in a peak shift of both the Si2p and O1s spectra for SiTi(20) to higher binding energy. This is evidence that the prolonged plasma treatment impacted the Si and O species near the catalyst surface. The shift to higher binding energy suggests that both Si and O have experienced a loss of electron density. This is the same sample which showed the emergence of an extra peak in the Raman spectra (Figure 4a), indicating that there may be a surface rearrangement which alters the way that Si and O interact on the catalyst surface. The shift of the Si2p spectra to higher BE represents a move towards pure SiO_2_ [44] and could signify a weakened interaction between the Si and Ti at the catalyst surface.

While plasma treatment of the hydrogenated SiTi(H) for 20 min resulted in an increase in Ti^3+^ content, it did not impact the Si2p or O1s spectra, where no peak shifts can be observed. This supports the observations from Raman spectroscopy (Figure 4b) and is further evidence that hydrogenation goes some way toward stabilising the Si–Ti interaction at the catalyst surface against plasma treatment, relative to the neat SiTi material.

**Figure 6:**
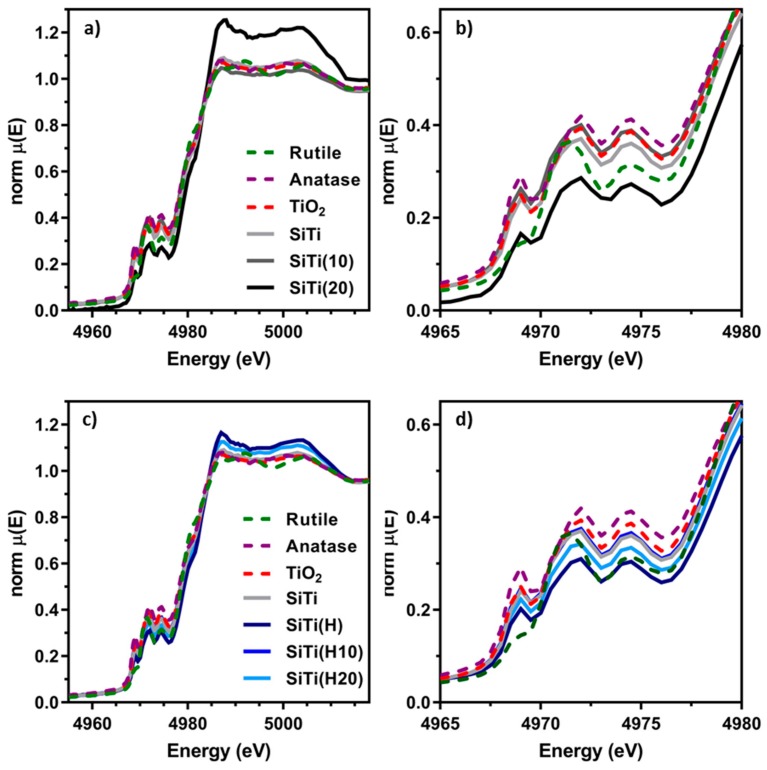
(**a**) Ti−K edge and (**b**) pre-edge XANES for FSP-synthesized SiTi before and after 10 and 20 min of plasma treatment. (**c**) Ti−K edge and (**d**) pre-edge XANES for FSP-synthesized SiTi after hydrogenation and/or 10–20 min of plasma treatment. Pure anatase, rutile, and FSP-prepared TiO_2_ controls are also included.

Ti K-edge XAS experiments were used to further understand the structural and chemical effects of hydrogenation and plasma processing on the SiTi. The X-ray absorption near edge spectra (XANES) for plasma treated and hydrogenated samples are shown in Figure 6 and are sensitive to local electronic structure, oxidation state, and local Ti-symmetry. The pre-edge feature of the Ti K-edge, representing 1 s → 3 d electronic transitions, is particularly sensitive to changes in local Ti symmetry and showcases notable structural changes across all samples. For reference, anatase and rutile are used for comparison purposes, along with SiTi and TiO_2_ synthesized via FSP without further chemical modification. Anatase exhibits well characterized pre-edge features at 4969 eV, 4972 eV, and 4974 eV, while rutile pre-edge features are positioned at 4971 eV and 4974 eV, with a small shoulder peak at 4969 eV. The materials synthesized by FSP exhibit very similar pre-edge features to anatase, suggesting the Ti atoms hold an anatase-type symmetry that is minimally influenced by the Si atoms, confirming the XRD and Raman analyses. Similarly, pre-edge feature positions for each hydrogenated SiTi material align with anatase, suggesting hydrogenation does not drastically alter the local Ti symmetry.

Unlike the hydrogenated samples, plasma treatment of the neat SiTi exhibits notable structural differences. SiTi(10) exhibits pre-edge features similar to anatase coupled with an additional shoulder at 4970 eV. The shoulder is likely indicative of the formation of five-fold coordinated Ti [45] rather than the formation of rutile, given that the lowest edge pre-edge feature is still present. The pre-edge features of SiTi(20) exhibit further perturbations away from the parent materials, where the lowest energy pre-edge feature is more similar to that of rutile, coupled with relative peak intensity changes. Examination of the pre-edge feature overall showcases that plasma treatment of SiTi materials influences the local symmetry of Ti, with longer plasma treatment times leading to more pronounced changes in material structure. This is expected to influence the catalytic properties of these materials.

Further examination of the XANES showcases additional changes in chemical and electronic structure of the SiTi materials with hydrogenation and plasma treatment. Overall, the shape of the XANES is very similar to that of anatase for all SiTi, consistent with our analysis of the pre-edge features above. Modest white line intensity increases are observed for the SiTi(H) and SiTi(H20) materials, while a much larger white line intensity is observed for SiTi(20). Increases in white line intensity indicate that the excited electron can access higher order molecular orbitals in the conduction band, almost always due to local electronic withdrawing effects of nearby atoms and/or a change in oxidation state. SiTi(20) also exhibits a large shift in E_0_ to higher energy, suggesting a bulk increase in Ti oxidation state. This is in line with the Raman analysis of SiTi(20), where peak broadening was attributed to a potential migration of surface or sub-surface oxygen into the bulk TiO_2_ material.

**Figure 7:**
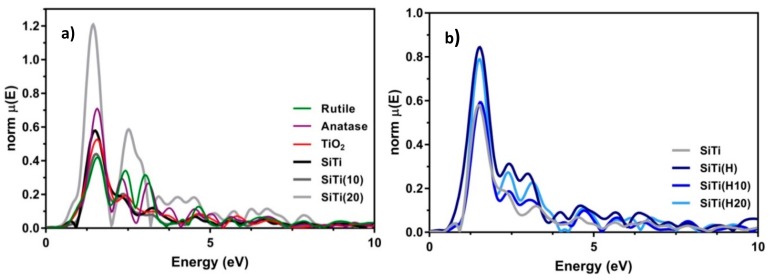
Ti K-edge EXAFS spectra of FSP-synthesized SiTi (**a**) before and (**b**) after hydrogenation and plasma treatment. Pure anatase, rutile, and FSP-prepared TiO_2_ controls are also included.

The extended X-ray absorption fine structure (EXAFS) spectra, presented in Figure 7, provides location structural information of the Ti–O and Ti–Ti nearest neighbour distances (NNDs). Note that all distances are not corrected for phase shift and are ~0.4 Å shorter than the actual NNDs. Both anatase and rutile exhibit at Ti–O NND 1.56 Å. Unmodified TiO_2_ synthesized by FSP exhibits a similar Ti–O NND, while the SiTi material has a reduced NND of 1.50 Å. For the hydrogenated materials, the Ti–O NNDs are 1.50, 1.55, and 1.52 Å for SiTi(H), SiTi(H10), and SiTi(H20), respectively. Note that these NNDs fall between the unmodified SiTi material and pure anatase, strongly suggesting that hydrogenation is affecting the local structure of the material. For the plasma treated materials, SiTi(10) exhibits a Ti–O NND of 1.51 Å, while a NND of 1.43 Å is observed for the SiTi(20). This large contraction of the Ti–O NND indicates that plasma treatment is having a profound effect on the structure of the catalysts, which in turn influences the catalytic properties.

Plasma induced structural changes are even more severely apparent when comparing longer NNDs corresponding to Ti–Ti distances of 2.32 Å and 3.12 Å in anatase. The starting SiTi FSP material exhibits significantly reduced features at these distances, which is expected when considering the nanoscale size of the particles. Upon plasma treatment, the Ti–Ti NNDs shift in the SiTi(10) sample, while a new feature appears in the SiTi(20) material. This feature has a maximum at 2.51 Å and a shoulder at 2.87 Å, which corresponds to an expansion and contraction of the relative Ti–Ti distance in anatase. The origin of these features cannot be determined at this time but must be due to a structural rearrangement of local Ti clusters due to the increased number of defects present in the material as a result of the prolonged plasma treatment. For the hydrothermally treated materials, the shift to longer NNDs is notable for all materials, indicative of a possible lattice expansion, although all samples retain the profile of an anatase-type structure. This suggests that the extended plasma treatment time for the SiTi material after hydrogenation has a less pronounced effect on Ti bonding, supporting the suggestion that hydrogenation assists in stabilising the TiO_2_ structure.

Plasma treatment of the mixed SiO_2_/TiO_2_ material before and after hydrogenation invokes modifications in the material structure and properties. The surface area and crystallinity of the SiTi materials is unaffected by up to 20 min of plasma treatment. Both the SiTi and SiTi(H) materials are altered by plasma treatment, according to the XPS analyses, with the reduction of Ti^4+^ to Ti^3+^ occurring among all samples, although the change brought about by plasma treatment was confined to the catalyst surface. XAS analyses demonstrate that chemical/structural changes occur in the SiTi materials, with more pronounced changes observed in the materials which did not undergo hydrogenation. In particular, there is an increase in the oxidation state (from XANES) and the emergence of new Ti–Ti features in SiTi(20). This same material exhibits a peak shift and broadening in Raman spectroscopy and a peak shift in the Si2p and O1s XPS spectra, which represents a change in the surface Si and O species, which may be related to a significant surface oxygen deficiency or internal stress. Hydrogenating the catalyst prior to conducting plasma treatment appears to be an effective way to limit these changes.

### 3.3. Photocatalytic Formic Acid Oxidation

**Figure 8:**
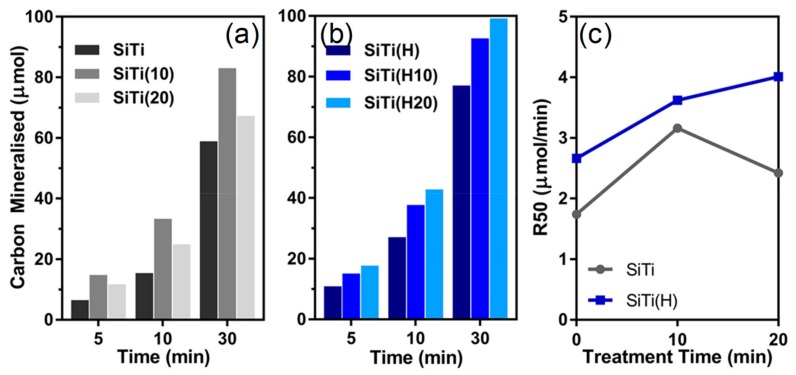
Carbon mineralization of (**a**) neat SiTi after plasma treatment, (**b**) hydrogenated SiTi after plasma treatment, and (**c**) the rate of photocatalytic formic acid oxidation (R_50_). Catalyst loading = 1 g/mL; suspension volume = 50 mL; suspension pH = 3 ± 0.1; formic acid loading = 100 µmol.

The ability of the mixed SiTi catalysts to activate oxygen was examined by monitoring the oxidation of formic acid under UV illumination. The mineralization of carbon and the rate of 50% formic acid oxidation (R_50_) were determined (Figure 8). Neat SiTi achieved a low rate of carbon mineralization, with only approximately 60% of the initial 100 μmol of formic acid oxidised to carbon dioxide after 30 min. As a result, the R_50_ of SiTi is 1.79 μmol/min. A 10 min plasma treatment of SiTi resulted in a sharp increase in the rate of carbon mineralization, especially in the first 10 min of activity, with SiTi(10) presenting an R_50_ of 3.16 μmol/min. This increase in the R_50_ can be attributed to the generation of surface Ti^3+^ species during plasma treatment. XPS revealed that the plasma treatment is effective in increasing the number of surface Ti^3+^ species, which likely contributes to the increase in activity for SiTi(10) relative to SiTi, as the Ti^3+^ was identified in a previous study as an active site for formic acid oxidation [19]. Furthermore, studies investigating the photocatalytic activity of doped TiO_2_ materials have shown that, while Ti^3+^ is a key component in oxygen activation, it is stable and remains active across multiple reaction cycles [46,47].

Increasing the plasma treatment to 20 min for SiTi(20) then caused a drop in performance, with the R_50_ of 2.42 μmol/min. The changes in material properties offer an explanation for this decrease in activity between 10 and 20 min of plasma treatment. While the plasma treatment did not affect the bulk TiO_2_ material, it resulted in significant changes to the catalyst surface. SiTi(20) was found with Raman and XPS to exhibit oxygen deficiencies and a decreased electron density for surface Si and O species. Thus, even though the surface Ti^3+^ content on SiTi(20) (6.5%) was higher than SiTi(10) (5.5%), the weakened interaction between Si/O and Ti at the catalyst surface outweighed this and decreased the photocatalytic activity of SiTi(20).

Hydrogenation of the mixed SiO_2_/TiO_2_ material increased its photocatalytic activity, where SiTi(H) achieves an R_50_ of 2.66 μmol/min compared to 1.79 μmol/min for neat SiTi. This increase in the rate of formic acid oxidation can be attributed to the reduction of Ti^4+^ to Ti^3+^ during the hydrogenation process. Plasma treatment of the hydrogenated sample resulted in a further increase in the rate of formic acid oxidation, with SiTi(H10) and SiTi(H20) achieving 92 and 99% conversion of formic acid in 30 min, respectively, corresponding to R_50_ values of 3.62 and 4.01 μmol/min. This improvement can be attributed to the greater Ti^3+^ presence on the surface of SiTi(H10) and SiTi(H20) and the stabilization of the SiTi material as a result of hydrogenation. As a result, the plasma treatment of the hydrogenated SiO_2_/TiO_2_ material results in further reduction of Ti^4+^ species present at or near the catalyst surface without impacting the Si–Ti interaction.

## 4. Conclusions

Mixed SiO_2_/TiO_2_ materials, synthesized via flame spray pyrolysis, were investigated as catalysts for the photocatalytic oxidation of formic acid. Hydrogenation of the SiTi materials was shown with EPR and XPS to result in the bulk reduction of Ti^4+^ to Ti^3+^. Plasma treatment of both the neat and hydrogenated SiTi materials resulted in the formation of increasing Ti^3+^ species at the catalyst surface, while the bulk material was left unaffected. The catalytic activity of the materials after hydrogenation and/or plasma treatment increased by approximately 40 to 130%, confirming that Ti^3+^ is integral in driving oxygen activation reactions. While higher Ti^3+^ content was related to increase photocatalytic activity, plasma treatment of the neat SiTi for 20 min was shown to result in a decreased Si–Ti interaction, which led to a drop in the rate of formic acid oxidation. This approach of introducing material defects presents a promising method for improving the performance of noble metal-free catalysts for oxygen activation reactions and could potentially be applied to advanced oxidation processes such as catalytic ozonation.

## Supplementary Materials

The following are available online at https://www.mdpi.com/1996-1944/12/17/2756/s1, Figure S1: Nitrogen adsorption-desorption isotherms of FSP-prepared SiTi before and after hydrogenation, Figure S2: Pore size distributions of FSP-prepared SiTi before and after hydrogenation, Figure S3: UV-Visible spectra of FSP-prepared SiTi before and after hydrogenation, measured using a BaSO_4_ reference, Figure S4: XRD reflections for FSP-synthesised SiTi before and after hydrogenation. Anatase (A) and Rutile (R) phases marked.
