# Supplementary material for: Plasma Treating Mixed Metal Oxides to Improve Oxidative Performance via Defect Generation
Source: Materials (Basel). 2019 Aug 27;12(17):2756. doi: 10.3390/ma12172756 (PMC6747793; doi:10.3390/ma12172756)
Supplement: Supplementary file 1 [file materials-12-02756-s001.pdf]

# Supplementary Materials: Plasma Treating Mixed Metal Oxides to Improve Oxidative Performance via Defect Generation

**Jonathan Horlyck [1], Alimatun Nashira [1], Emma Lovell [1], Rahman Daiyan [1], Nicholas Bedford [1], Yuexing Wei [2], Rose Amal [1], and Jason Scott [1,*]**

[1] School of Chemical Engineering, the University of New South Wales, Sydney, NSW 2052, Australia
[2] School of Energy and Environment, Southeast University, Nanjing 210096, China
* Correspondence: jason.scott@unsw.edu.au
Received: 1 August 2019; Accepted: 26 August 2019; Published: 27 August 2019

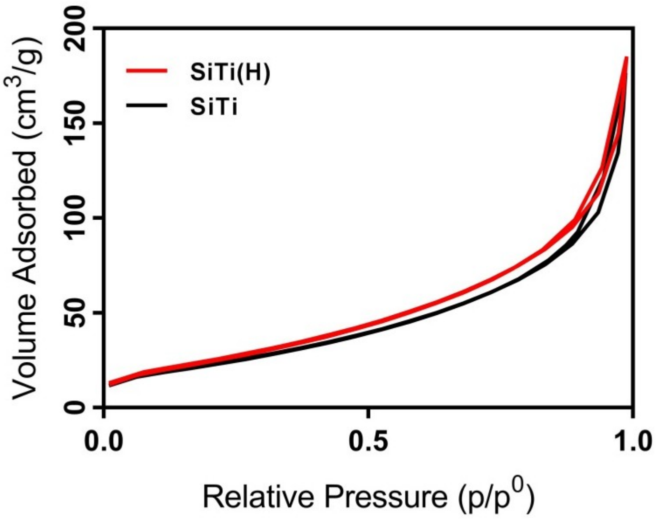


**Figure S1.** Nitrogen adsorption-desorption isotherms of FSP-prepared SiTi before and after hydrogenation.

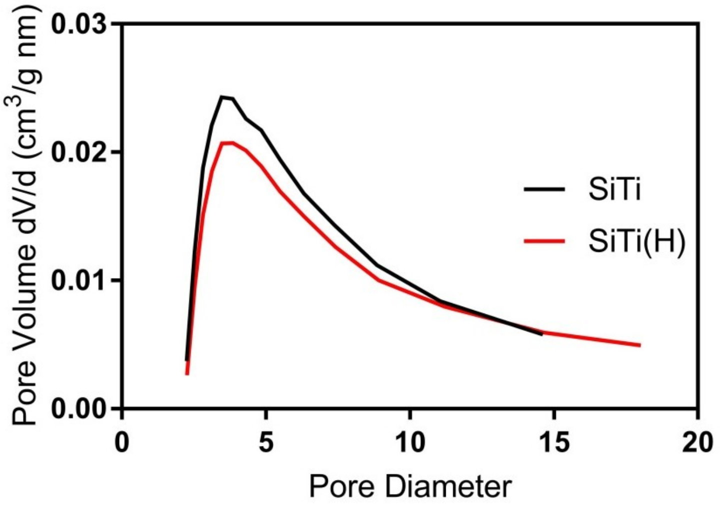


**Figure S2.** Pore size distributions of FSP-prepared SiTi before and after hydrogenation.

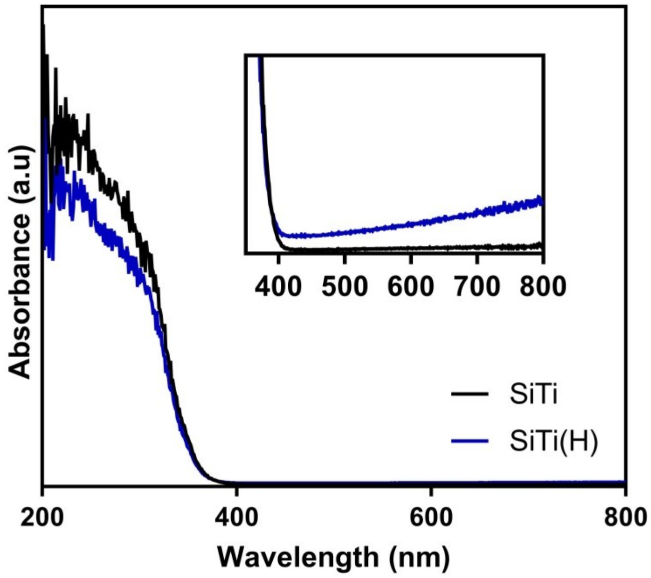


**Figure S3.** UV-Visible spectra of FSP-prepared SiTi before and after hydrogenation, measured using a $BaSO_4$ reference.

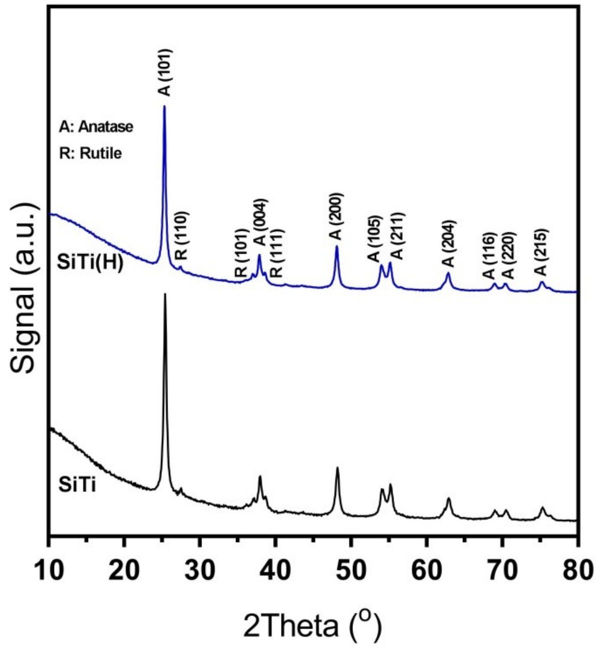


**Figure S4.** XRD reflections for FSP-synthesised SiTi before and after hydrogenation. Anatase (A) and Rutile (R) phases marked.
